# Insights on Distinct Left Atrial Remodeling Between Atrial Fibrillation and Heart Failure With Preserved Ejection Fraction

**DOI:** 10.3389/fcvm.2022.857360

**Published:** 2022-04-26

**Authors:** Jen-Yuan Kuo, Xuanyi Jin, Jing-Yi Sun, Sheng-Hsiung Chang, Po-Ching Chi, Kuo-Tzu Sung, Greta S. P. Mok, Chun-Ho Yun, Shun-Chuan Chang, Fa-Po Chung, Ching-Hsiang Yu, Tung-Hsin Wu, Chung-Lieh Hung, Hung-I Yeh, Carolyn S. P. Lam

**Affiliations:** ^1^Division of Cardiology, Department of Internal Medicine, MacKay Memorial Hospital, Taipei, Taiwan; ^2^Department of Medicine, Mackay Medical College, New Taipei City, Taiwan; ^3^Mackay Medicine, Nursing and Management College, Taipei, Taiwan; ^4^National Heart Centre Singapore, and Duke-National University of Singapore, Singapore, Singapore; ^5^Department of Cardiology, University of Groningen, University Medical Centre Groningen, Groningen, Netherlands; ^6^Department of Biomedical Imaging and Radiological Sciences, National Yang Ming University, Taipei, Taiwan; ^7^Division of Cardiology, Department of Internal Medicine, Taoyuan General Hospital, Taoyuan, Taiwan; ^8^Biomedical Imaging Laboratory, Department of Electrical and Computer Engineering, Faculty of Science and Technology, University of Macau, Macao, Macao SAR, China; ^9^Department of Radiology, MacKay Memorial Hospital, Taipei, Taiwan; ^10^Holistic Education Center, Mackay Medical College, New Taipei City, Taiwan; ^11^Institute of Clinical Medicine and Cardiovascular Research Center, National Yang-Ming University, Taipei, Taiwan; ^12^Heart Rhythm Center and Division of Cardiology, Department of Medicine, Taipei Veterans General Hospital, Taipei, Taiwan; ^13^Institute of Biomedical Sciences, Mackay Medical College, New Taipei City, Taiwan

**Keywords:** atrial fibrillation, heart failure with preserved ejection fraction, strain, multi-detector computed tomography, left atrial remodeling, left atrial wall

## Abstract

**Background:**

Heart failure with preserved ejection fraction (HFpEF) and atrial fibrillation (AF) commonly coexist with overlapping pathophysiology like left atrial (LA) remodeling, which might differ given different underlying mechanisms.

**Objectives:**

We sought to investigate the different patterns of LA wall remodeling in AF vs. HFpEF.

**Methods:**

We compared LA wall characteristics including wall volume (LAWV), wall thickness (LAWT), and wall thickness heterogeneity (LAWT[SD]) and LA structure, function among the controls (without AF or HFpEF, *n* = 115), HFpEF alone (*n* = 59), AF alone (*n* = 37), and HFpEF+AF (*n* = 38) groups using multi-detector computed tomography and echocardiography.

**Results:**

LA wall remodeling was most predominant and peak atrial longitudinal strain (PALS) was worst in HFpEF+AF patients as compared to the rest. Despite lower E/e' (9.8 ± 3.8 vs. 13.4 ± 6.4) yet comparable LA volume, LAWT and PALS in AF alone vs. HFpEF alone, LAWV [12.6 (11.6–15.3) vs. 12.0 (10.2–13.7); *p* = 0.01] and LAWT(SD) [0.68 (0.61–0.71) vs. 0.60 (0.56–0.65); *p* < 0.001] were significantly greater in AF alone vs. HFpEF alone even after multi-variate adjustment and propensity matching. After excluding the HFpEF+AF group, both LAWV and LAWT [SD] provided incremental values when added to PALS or LAVi (all p for net reclassification improvement <0.05) in discriminating AF alone, with LAWT[SD] yielding the largest C-statistic (0.78, 95% CI: 0.70–0.86) among all LA wall indices.

**Conclusions:**

Despite a similar extent of LA enlargement and dysfunction in HFpEF vs. AF alone, larger LAWV and LAWT [SD] can distinguish AF from HFpEF alone, suggesting the distinct underlying pathophysiological mechanism of LA remodeling in AF vs. HFpEF.

## Introduction

Heart failure with preserved ejection fraction (HFpEF) and atrial fibrillation (AF) are frequently coexisting conditions that are growing in prevalence and share common predisposing factors such as older age, hypertension, and obesity (1–3). It remains a diagnostic dilemma to discriminate HFpEF from AF due to convergent clinical features (e.g., breathlessness, effort intolerance). Yet, to accurately distinguish one condition from the other is important since their treatments may differ, partly due to their potentially distinct underlying pathophysiology.

Beyond sharing predisposing factors, left atrial (LA) remodeling is also a key characteristic that is common to both HFpEF and AF. In HFpEF, LA remodeling is thought to be secondary to an increase in LA pressure and potential intrinsic LA myopathy; however, in AF, LA remodeling is thought to be triggered by ischemia, inflammation, or dilation and perpetuated by tachycardia-induced remodeling (4, 5). The common result in both conditions is an enlarged, dysfunctional left atrium, which is difficult to differentiate by conventional imaging modalities. Compared to these traditional methods, more advanced imaging techniques such as multi-detector computed tomography (MDCT) with higher spatial resolution or echocardiographic strain imaging are known to provide additional information about LA structure and function beyond conventional imaging modalities, which may provide novel insights into LA remodeling and distinct patterns of LA remodeling in AF vs. HFpEF (6).

Therefore, we aimed to compare the LA structure, function, and wall characteristics among four groups of patients, namely the controls without HFpEF or AF, patients with HFpEF alone, patients with AF alone, and patients with both HFpEF and AF, by using a combination of MDCT and comprehensive echocardiography.

## Methods

### Study Population

**Figure 1:**
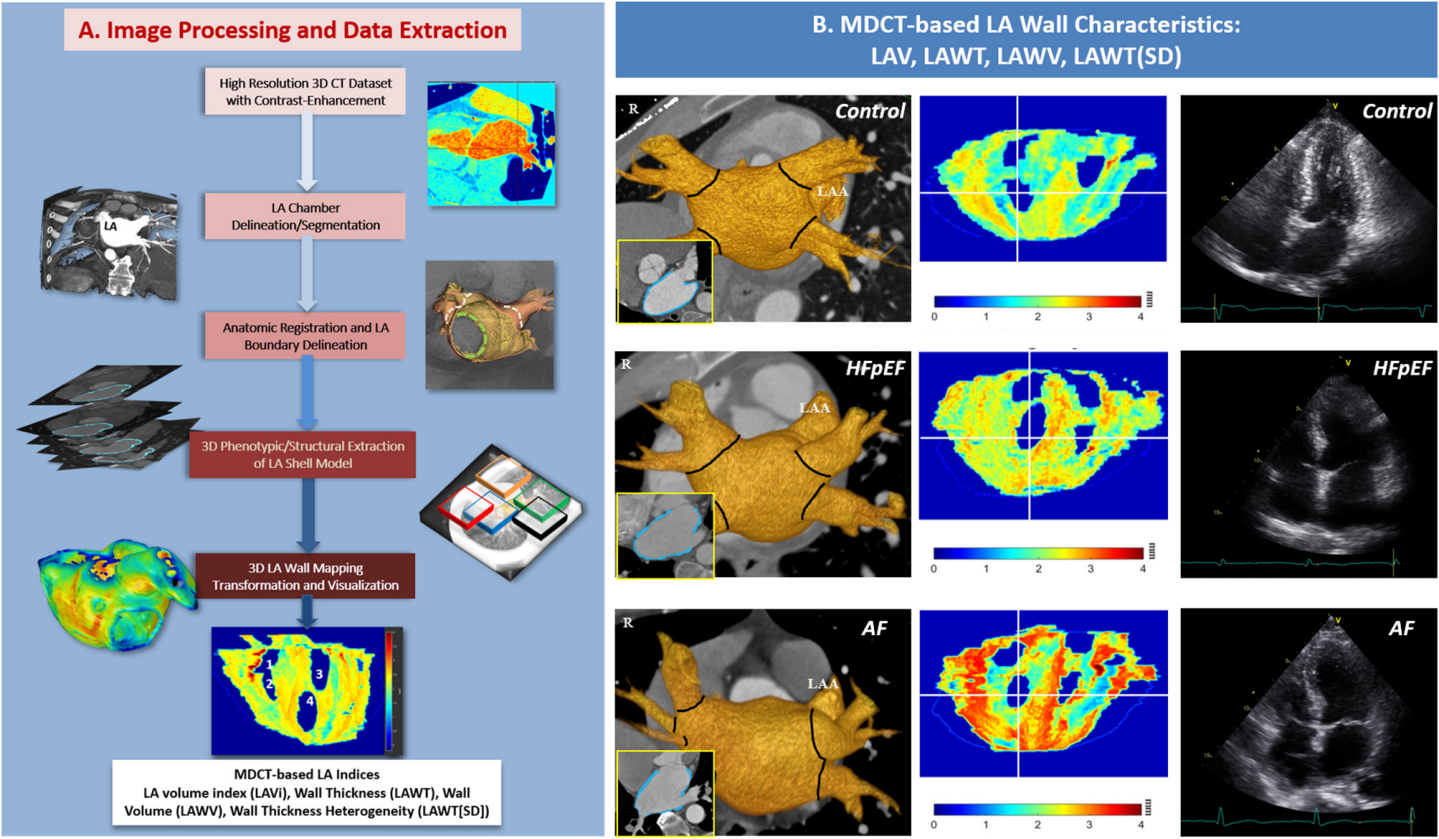
Five major steps in the LA mapping workflow for LA wall indices in the current study: (1) LA delineation, (2) inner boundary segmentation, (3) outer boundary segmentation, (4) wall mass calculation, (5) three-dimensional (3D) projection map, and display of the LAWT (SD). The LAWT in each dataset was expressed by different colors as a visual projection map together with four PVs orifices. Nos. 1, 2, 3, and 4 represent the right superior, right inferior, left superior, and left inferior PVs, respectively **(A)**. Cardiac CT images from 3 representative individuals from the Control, HFpEF, and AF groups **(B)**. LA, left atrial; LAWT, left atrial wall thickness; LAWT (SD), left atrial wall thickness heterogeneity; PV, pulmonary vein; MDCT, multi-detector computed tomography; HFpEF, heart failure with preserved ejection fraction; AF, atrial fibrillation.

Patients who were referred to the MacKay Memorial Hospital (Taipei, Taiwan) from a cardiovascular imaging core laboratory for clinical evaluation of ischemic heart disease or AF ablation between January 2009 and December 2014 were retrospectively identified through a medical record review. HFpEF was defined as prior HF hospitalization and a left ventricular ejection fraction (LVEF) of >50%. Adjudication of HF hospitalization was defined by two cardiologists with typical symptoms (dyspnea, breathlessness or ankle swelling) and signs of pulmonary congestion or edema by Chest X-ray. These were followed by natriuretic peptide cutoffs (BNP: 100 pg/ml, NT-proBNP: 300 pg/ml) proposed by 2021 ESC HF guideline to exclude those without acute HF. AF was ascertained by a history of paroxysmal or persistent AF or those referred for evaluation of catheter ablation for AF. Diagnosis of AF was made by standard 12-lead electrocardiogram (ECG), or ECG tracing of ≥30 s showing AF rhythm from 24-h Holter monitoring. Days of diagnosed paroxysmal AF in our AF patients was 181.1 ± 71.2 days. Each patient underwent comprehensive clinical evaluation, laboratory tests, Holter monitoring, dual-source cardiac MDCT scans, and comprehensive echocardiography. Imaging (including echocardiography and MDCT) protocol was conducted during sinus rhythm prior to AF ablation (if ablation therapy was delivered). Patients with significant valvular heart disease, severe pulmonary hypertension, known cardiomyopathy, permanent AF, and those with pacemaker implantation were excluded. Thus, a total of 249 patients were included and divided into four groups, namely the HFpEF alone (*n* = 59), AF alone (*n* = 37), HFpEF+AF (*n* = 38, both HFpEF and AF), and controls (*n* = 115 with neither HFpEF nor AF) groups (Figure 1B). The study protocol was approved by the Institutional Review Board of MacKay Memorial Hospital (19MMHIS213e).

### Computed Tomography Image Acquisition Protocol and LA Wall Characteristics Analysis

Each patient underwent cardiac MDCT using a dual-source, high-resolution CT system (Siemens Medical Systems, Forchheim, Germany). The step-by-step protocols for scanning, image acquisition, reconstruction, and subsequent LA wall characteristics analysis using our validated LA wall-mapping software have been previously published (Supplementary Materials) (6). In brief, the LA wall-mapping application enables the automatic isocenter setting of the LA chamber, LA inner-surface, and outer-boundary segmentation, and facilitates delineation after manual delineation of the pulmonary veins. The LA wall volume (LAWV) was derived by integrating the total number of voxels of the LA tissue, which yielded the total LA volume contained within the outer boundaries, minus the chamber volume within the inner surface of LA. Wall thickness was defined as the shortest distance between the outer boundaries and the inner surfaces, with the representative LA wall thickness (LAWT) of any individual calculated as the average distance within the entire LA region. LAWT heterogeneity was assessed as the variations [expressed as standard deviation (SD); thus, LAWT(SD)] in the LAWT within the entire LA region measured by the LA wall-mapping program (Figure 1A). Finally, the LA volume (LAV) was calculated by integrating the number of voxels contained within the inner surfaces, with the LAV further indexed (LAVi) to the individual's body surface area.

### Echocardiography

Each patient underwent a complete transthoracic echocardiography exam within 30 days of MCDT, using a Vivid 7 system (GE Healthcare, Chicago, IL, USA), equipped with a 2- to 4-MHz transducer (M4S). All echocardiographic image acquisition and measurements were performed on the basis of recent guidelines recommended by the American Society of Echocardiography (7). All echocardiographic measurements, including two-dimensional speckle-tracking echocardiographic analysis, were performed off-line using EchoPAC version 6.1.2 (GE Vingmed, Horten, Norway). The protocol for the same was previously described in detail (8). LV end-diastolic volume (EDV) and LVEF were measured using the biplane Simpson method. Pulse wave Doppler was applied to record the trans-mitral inflow early (E) and late diastolic (A) filling velocities, deceleration time (DT), and isovolumic relaxation time. Peak tricuspid regurgitation (TRV) velocity was obtained using continuous wave Doppler. Peak mitral annular systolic (TDI-s′) and early diastolic (TDI-e′) velocities were obtained using Tissue Doppler imaging and were averaged from the septal and lateral mitral annular sides, respectively. The ratio of mitral inflow E over A (E/A) and the average E/e' ratio (E/e') were calculated accordingly, with E/e' used for estimating the LV filling condition. The LV global longitudinal strain (LVGLS) was averaged from the peak longitudinal strain across 18 LV segments obtained from the LV apical 4-, 2-, and 3-chamber views. Global peak atrial longitudinal strain (PALS) and global longitudinal LA strain rate during the reservoir (SRs), conduit (SRe), and booster (SRa) phases were averaged from all LA segments of the apical 4- and 2-chamber views, respectively.

### Statistical Analyses

A *priori* sample size calculation, based on the LAWT differences, was performed using the Power and Sample Size software version 08 (NCSS, LLC, Kaysville, UT, USA). All data were expressed as means ± standard deviation (SD) or medians ±inter-quartile range (IQR) for continuous variables and percentage or frequency for categorical variables. Comparative analysis for parametric continuous variables was performed using a one-way analysis of variance or an independent *t-*test and for non-parametric continuous variables using the Kruskal–Wallis test or a Mann-Whitney U test. Categorical variables were compared using either the Fisher's exact test or the χ^2^ test with a Yates correction, as appropriate. Furthermore, we compared LA wall characteristics in control vs. HFpEF alone and control vs. AF alone after stratified based on median value of MDCT-based LAVi (<40 vs. ≥40 ml/m^2^), respectively. Associations of various LA wall indices with clinical co-variates were determined by forward stepwise selection including age, sex, body mass index (BMI), lipid profile, medical history, estimated glomerular filtration rate (eGFR) calculated by MDRD equation, high sensitivity C-reactive protein (hs-CRP), and E/e'. To further elaborate the LA wall features in patients with isolated AF, C-statistic was used to examine the performance of LA wall indices in distinguishing AF from HFpEF and controls after excluding the HFpEF+AF group. Moreover, comparisons were made again after 1:1 matching (controls and HFpEF vs. AF) for key clinical co-variates. Net reclassification index was used to assess the incremental value of LA wall indices in re-classifying AF in addition to LAVi and PALS.

The linear correlations between the LA wall indices and echocardiographic parameters were determined using the Pearson correlation coefficient (r) and maximal information criteria (MIC). The differences in absolute values between the MIC and the squared Pearson correlation (*r*^2^) were used to determine the non-linear correlation (i.e., MIC-r^2^ ≥ 0.1). Subsequently, the cluster model was constructed to assess the similarity between LA wall indices and echocardiographic parameters, and the details of constructing this cluster model can be found in the Supplementary Material. All analyses were performed using STATA 14.0 software (Stata Corp, College Station, TX, USA). All *p*-values were two-tailed, with *p* < 0.05 considered statistically significant.

## Results

### Baseline Demographics and Echocardiographic Characteristics

Table 1 summarizes the baseline clinical, laboratory, and echocardiographic characteristics of all patients. Patients with HFpEF+AF demonstrated the highest comorbidity burdens, highest BNP levels, worst renal function based on the eGFR, the most advanced cardiac remodeling and worst LV, LA function as compared to the rest of patients. Despite similar age and BMI, patients with AF alone had a higher burden of hypertension and diabetes as compared to control participants. Whereas, patients with HFpEF alone were more likely to be older, female, and present with more comorbidities, including hypertension, diabetes, and coronary artery disease, and higher BMI as compared to control participants. Similarly, patients with HFpEF alone were more likely to be older, female, and present with diabetes and higher B-type natriuretic peptide (BNP) level as compared to the patients with AF alone.

**Table 1:** Baseline characteristics and conventional echocardiographic parameters of the study subjects.

	**Controls**	**HFpEF**	**AF**	**HFpEF +AF**	***Overall p*** **-value**
	**(*n* = 115)**	**(*n* = 59)**	**(*n* = 37)**	**(*n* = 38)**	
***Baseline Characteristics***					
Age (years)	61.4 ± 10.8	69.1 ± 8.1*	59.7 ± 10.3^†^	68.1 ± 11.9*^#^	<0.001
Female sex, (%)	34	61.0*	40.5^†^	47.4	0.007
Systolic blood pressure, mmHg	128.4 ± 15.7	131.6 ± 15.5	125.5 ± 17.8	130.7 ± 20.3	0.31
Diastolic blood pressure, mmHg	76.3 ± 9.7	73.9 ± 9.0	74.5 ± 10.1	73.1 ± 11.3	0.25
Heart rate, beats/min	76.1 ± 11.6	82.2 ± 16.5*	82.1 ± 16.3	78.4 ± 12.4	0.02
BMI, kg/m^2^	24.7 ± 3.4	26.3 ± 3.8*	25.8 ± 4.2	26.4 ± 4.4	0.018
Smoking, (%)	18.3	17	16.2	31.6	0.25
Hypertension, (%)	28.7	69.5*	73.0*	84.2*	<0.001
Diabetes, (%)	20	49.2*	35.1	52.6*	<0.001
Hyperlipidemia, (%)	38.3	49.2	40.5	55.3	0.23
Coronary artery disease, (%)	11.3	15.3	13.5	36.8*^†^^#^	0.003
***Laboratory Data***					
HbA1c, % (*n* = 196)	5.96 ± 0.66	6.48 ± 1.42	6.54 ± 1.22*^†^	6.51 ± 1.67	0.016
Fasting glucose, mg/dl	108.8 ± 19.9	126.7 ± 55.2	122.6 ± 41.1*	140.5 ± 66.1*	<0.001
Cholesterol, mg/dl	185.0 ± 39.8	189.9 ± 43.5	194.7 ± 42.0	166.8 ± 39.4	0.82
Triglyceride, mg/dl	119.0 ± 67.0	139.9 ± 70.3	157.8 ± 133.1	151.6 ± 80.0	0.037
LDL-c, mg/dl	111.6 ± 38.2	116.3 ± 38.7	111.2 ± 41.1	111.6 ± 33.0	0.88
HDL-c, mg/dl	50.2 ± 13.4	46.7 ± 14.6	46.5 ± 10.8	44.4 ± 13.6	0.096
eGFR, ml/min/1.73 m^2^	84.4 ± 21.7	77.1 ± 29.0*	83.2 ± 24.0	59.7 ± 25.9*^†^^#^	<0.001
BNP [median, IQR], pg/ml (*n* = 172)	19.9 [10.3–36]	120 [46–352]*	61 [35.5–145]*^†^	482 [176–915]*^†^^#^	<0.001
hs-CRP [median, IQR], mg/dl (*n* = 197)	0.11 [0.045–0.35]	0.35 [0.11–1.71]*	0.63 [0.21–2.09]*	1.42 [0.34–2.25]*^†^	<0.001
***Cardiac Structure by echocardiography***					
Septal wall thickness, cm	0.90 ± 0.15	1.02 ± 0.18*	0.93 ± 0.13	1.02 ± 0.21*	<0.001
Posterior wall thickness, cm	0.91 ± 0.13	1.03 ± 0.18*	0.95 ± 0.14	1.04 ± 0.16*	<0.001
LV internal diameter, cm	4.62 ± 0.41	4.68 ± 0.55	4.71 ± 0.43	4.73 ± 0.60	0.55
LV mass index, gm/m^2^	75.5 ± 15.9	92.9 ± 25.9*	79.7 ± 18.5^†^	93.3 ± 25.0*^#^	<0.001
***LV Function***					
LVEF, %	63.5 ± 7.8	60.6 ± 8.4	61.7 ± 7.7	62.8 ± 10.5	0.16
E/A	0.97 ± 0.33	1.03 ± 0.48	1.68 ± 0.90*^†^	1.75 ± 1.05*^†^	<0.001
DT, msec	224.6 ± 48.4	244.1 ± 78.6	217.8 ± 63.9	212.2 ± 60.5	0.05
E/e'	8.7 ± 2.9	13.4 ± 6.4*	9.8 ± 3.8^†^	16.4 ± 11.2*^#^	<0.001
TDI-e', cm/s	7.6 ± 1.9	6.3 ± 1.5*	7.8 ± 2.1*^†^	6.3 ± 2.0*^#^	<0.001
TDI-s', cm/s	7.6 ± 1.5	6.8 ± 1.6*	7.3 ± 1.5*	5.9 ± 1.4*^†^^#^	<0.001
TRV, m/s	2.2 ± 0.3	2.9 ± 0.4*	2.7 ± 0.4*^†^	3.2 ± 0.4*^†^^#^	<0.001
LVGLS, %	−19.1 ± 3.2	−17.0 ± 4.5*	−18.6 ± 2.6	−14.2 ± 3.6*^†^^#^	<0.001
***LA Function***					
PALS, %	35.0 ± 7.3	27.4 ± 7.7*	28.7 ± 9.5*	23.8 ± 7.3*^#^	<0.001
SRs *(Reservoir)*, %	1.52 ± 0.35	1.20 ± 0.29*	1.24 ± 0.34*	1.04 ± 0.25*	<0.001
SRe (Conduit), %	−1.48 ± 0.44	−1.07 ± 0.40*	−1.13 ± 0.35*	−0.89 ± 0.28*	<0.001
SRa (Booster pump), %	−1.93 ± 0.48	−1.13 ± 0.40*	−1.04 ± 0.48*	−0.93 ± 0.41*	<0.001

Data were expressed as the mean ± SD, (%), or median [IQR]. *p <0.05 vs. Controls;^†^p < 0.05 vs. HFpEF; ^#^p <0.05 vs. AF. AF, atrial fibrillation; BMI, body mass index; BNP, brain natriuretic peptide; eGFR, estimated glomerular filtration rate; DT, deceleration time; E/e', ratio of the early transmitral hemodynamic Doppler E velocity divided by the TDI-e'; Hb1Ac, hemoglobin A1c; HDL-c, high-density lipoprotein-cholesterol; HFpEF, heart failure with a preserved ejection fraction; hs-CRP, high sensitivity C-reactive protein; IQR, interquartile range; LA, left atrial; LDL-c, low-density lipoprotein-cholesterol; LV, left ventricular; LV EF, LV ejection fraction; LVGLS, LV global longitudinal strain; PALS, global longitudinal LA strain; SR, strain rate; TDI-e', average of mitral annular early relaxation velocity from septal and lateral sites by tissue Doppler imaging; TDI-s', average of mitral annular contraction velocity from septal and lateral sites by tissue Doppler imaging; TRV, peak tricuspid regurgitant velocity.

Patients with HFpEF alone and HFpEF+AF showed greater LV mass index as compared to both control and patients with AF alone; whereas LV mass index was similar between control and patients with AF alone (Table 1). As expected, LV function evaluated by LVGLS or TDI-s' was worse in patients with HFpEF+AF as compared to the rest of the patients, despite similar LVEF among the four groups. Although LVGLS or TDI-s' was worse in patients with HFpEF alone, but similar in patients with AF alone as compared to control patients. Notably, LV diastolic dysfunction presented with reduced TDI-e', increased E/e' ratio and higher pulmonary arterial pressure estimated by TRV, was more advanced in the patients with HFpEF alone and HFpEF+AF as compared to the patients with AF alone.

### LA Structure, Function and LA Wall Characteristics

Table 2 shows the LA wall characteristics among the four groups. Reproducibility of the all LA wall indices in 30 random study participants are presented in Supplementary Table 1. All LA wall indices [LAWV, 15.4 (13.5~18.2) ml; LAWT, 2.10 (2.00~2.27) mm; LAWT (SD), 0.64 (0.57~0.71) and LAVi [62.3 (48.6~81.0) ml/m^2^] was largest, and PALS (23.8 ± 7.3 %) as well as phasic LA strain rate components (Table 2) were worst in patients with HFpEF+AF group, indicating the most advanced LA remodeling and LA global dysfunction in this group. In contrast, all LA wall indices [LAWV, 10.7 (9.5~12.1) ml; LAWT, 1.91 (1.81~2.02) mm; LAWT (SD), 0.58 (0.55~0.61) and LAVi [LAVi, 33.6 (27.3~39.5) ml/m^2^] were smallest, PALS (35 ± 7.3%) as well as LA phasic strain rate components were largest in control patients (Table 2). The LAVi (LAVi: 44.4 [37.2–52.1] vs. 45.8 [34.6~51.4] ml/m^2^; *p* = 0.46 in HFpEF vs. AF alone), all LA global phasic function (PALS, SRs, SRe, and SRa; Table 1) and LAWT [2.02 (1.91–2.12) vs. 2.06 (1.99–2.22) mm; *p* > 0.05 in HFpEF vs. AF alone] were similar in the patients with HFpEF alone vs. AF alone. Despite many similarities of LAVi, LAWT and global LA function, the LAWV and LAWT (SD) were significantly larger in patients with AF alone as compared to patients with HFpEF alone [LAWV:12.6 (11.6–15.3) vs. 12.0 (10.2–13.7) ml, *p* = 0.01; and LAWT(SD): 0.68 (0.61–0.71) vs. 0.60 (0.56–0.65), *p* <0.001, respectively]. Notably, LAWT(SD) was substantially greater in patients with AF alone than in patients with HFpEF alone even after adjusting for key clinical co-variates and E/e' (Table 2) or after 1:1 propensity matching based on key baseline co-variates in control patients and patients with HFpEF alone vs. patients with AF alone (Supplementary Table 2) (Table 3).

**Table 2:** Baseline atrial structure and LA wall indices of the study subjects by MDCT.

	**Controls (*****n*** **= 115)**		**HFpEF (*****n*** **= 59)**		**AF (*****n*** **= 37)**		**HFpEF + AF (*****n*** **= 38)**		***P*** **-value^‡^**
	**Median [IQR]**	**SE**	**Median [IQR]**	**SE**	**Median [IQR]**	**SE**	**Median [IQR]**	**SE**	
**Non-adjusted median [IQR]**									
LAVi, ml/m^2^	33.6 [27.3–39.5]	0.77	44.4 [37.2–52.1]*	1.83	45.8 [34.4–52.1]*	2.12	62.3 [48.3–81.3]*^†^^#^	4.6	<0.001
LAWV, ml	10.7 [9.50–12.1]	0.19	12.0 [10.2–13.7]*	0.32	12.6 [11.6–15.6]*^†^	0.49	15.4 [13.3–18.5]*^†^^#^	0.77	<0.001
LAWT, mm	1.91 [1.81–2.02]	0.02	2.02 [1.91–2.12]*	0.03	2.06 [1.93–2.25]*	0.04	2.10 [2.00–2.28]*^†^	0.03	<0.001
LAWT (SD)	0.58 [0.55–0.61]	0.004	0.60 [0.56–0.65]	0.015	0.68 [0.61–0.71]*^†^	0.013	0.64 [0.57–0.72]*	0.022	<0.001
**Multi-variate adjusted median [IQR]**									
LAVi, ml/m^2^	34.5 [27.4–41.0]	0.77	44.7 [37.4–51.1]*	1.83	45.6 [34.6–58.6]*	2.12	63.9 [48.6–82.0]*^†^#	4.6	<0.001
LAWV, ml	11.1 [9.83–12.6]	0.18	12.3 [10.5–13.7]*	0.31	13.3 [11.8–16.2]*^†^	0.49	15.4 [13.5–17.4]*^†^^#^	0.77	<0.001
LAWT, mm	1.92 [1.81–2.04]	0.02	2.05 [1.92–2.14]*	0.03	2.04 [1.86–2.24]*	0.04	2.10 [2.00–2.25]*^†^	0.03	<0.001
LAWT (SD)	0.58 [0.55–0.62]	0.006	0.61 [0.56–0.65]	0.014	0.69 [0.61–0.71]*^†^	0.011	0.63 [0.57–0.72]*	0.019	<0.001

*Data were expressed as median [IQR].^‡^Statistical analyses were performed by non-parametric tests with pairwise Wilcox test (Kruskal–Wallis rank sum test)*.

*Multi-variate models were adjusted for age, sex, BMI, hypertension, diabetes, coronary artery disease, hyperlipidemia, eGFR and E/e'*.

*p <0.05 vs. Controls;^†^p <0.05 vs. HFpEF; ^#^p <0.05 vs. AF. LA, left atrial; LAVi, left atrial volume index; LA WT, left atrial wall thickness; LA WT (SD), left atrial wall thickness heterogeneity; LA WV, left atrial wall volume.

**Table 3:** Comparisons of atrial structure and LA wall indices of the study subjects by MDCT after matching.

	**Controls and HFpEF (*****n*** **= 37)**		**AF (*****n*** **= 37)**		***P*** **-value^‡^**
	**Median [IQR]**	**SE**	**Median [IQR]**	**SE**	
LAVi, ml/m^2^	38.69 [32.41–47.90]	2.48	45.83 [34.59–51.44]	2.12	0.15
LA WV, ml	11.5 [10.69–12.98]	0.30	12.57 [11.63–15.34]	0.48	0.006
LA WT, mm	2.0 [1.86–2.08]	0.04	2.06 [1.99–2.22]	0.04	0.07
LA WT (SD)	0.58 [0.56–0.64]	0.012	0.68 [0.61–0.71]	0.013	<0.001

*Data were expressed as median [IQR: 25^th^ ~ 75^th^]. Variables used for matching included age, sex, BMI, hypertension, diabetes, coronary artery disease, hyperlipidemia, eGFR and E/e'*.

*^‡^Statistical analyses were performed by non-parametric Mann-Whitney U test*.

### Clinical Correlates of LA Wall Indices by MDCT

By forward stepwise selection, LAVi enlargement was independently associated with greater BMI and higher E/e' (all *p* <0.05), and LAWV enlargement was independently associated with greater BMI and lower eGFR (all *p* < 0.05). Increase of LAWT was independently associated with older age and presence of hypertension (all *p* <0.05). Furthermore, greater LAWT(SD) was independently associated with the male sex, higher hs-CRP level, and lower high-density lipoprotein-cholesterol level (all *p* <0.05).

### Correlation Between LA Wall Indices by MDCT and Echocardiographic Parameters

**Figure 2:**
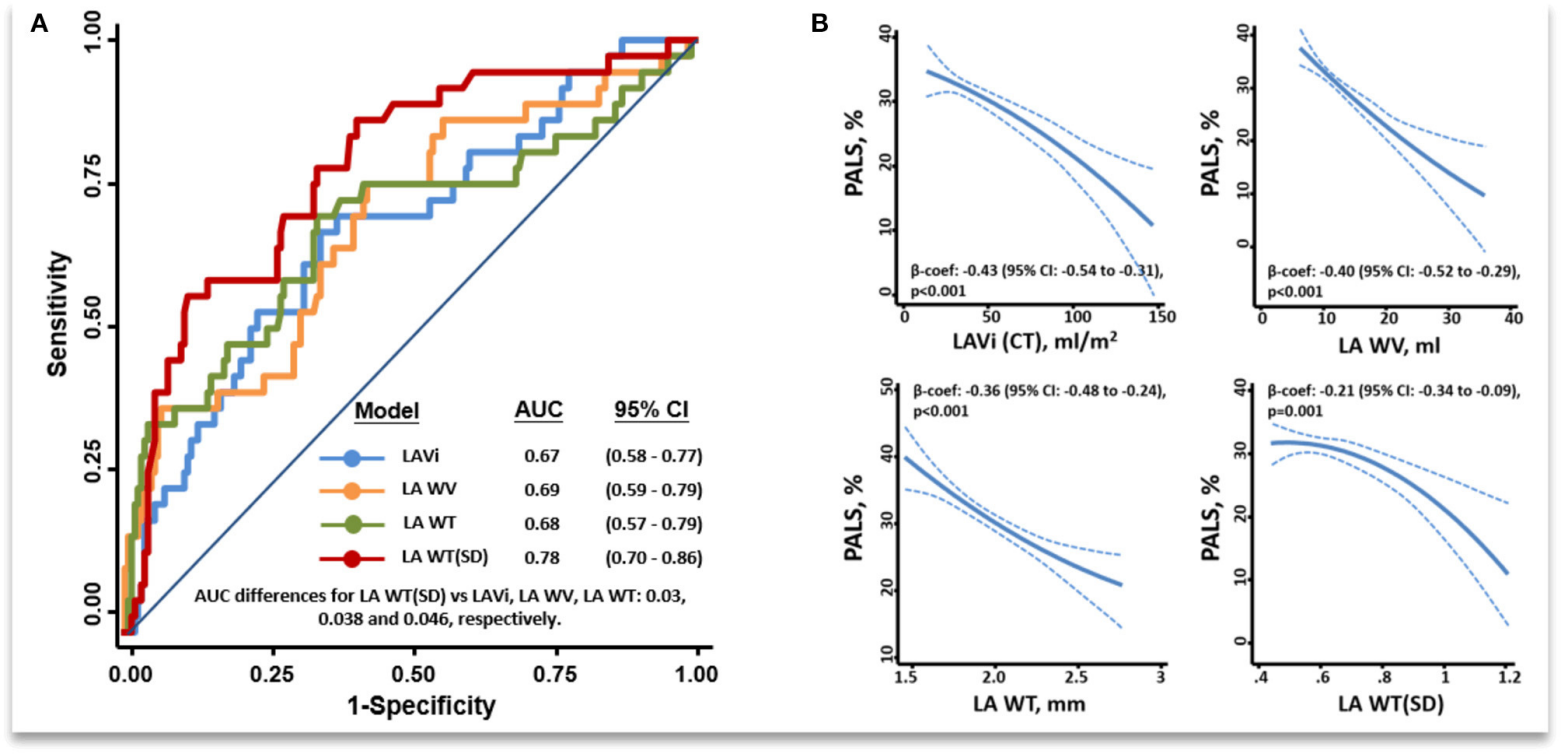
ROC among all LA wall indices in discriminating isolated AF from control and HFpEF after excluding patients with both HFpEF and AF (final *n* = 211) **(A)**. Fitting curves showing inverse associations between a greater unfavorable remodeling of the various MDCT LA wall indices and PALS **(B)**. ROC, receiver operating characteristic curve; PALS, Peak atrial longitudinal strain; other abbreviations as Figure 1.

**Figure 3:**
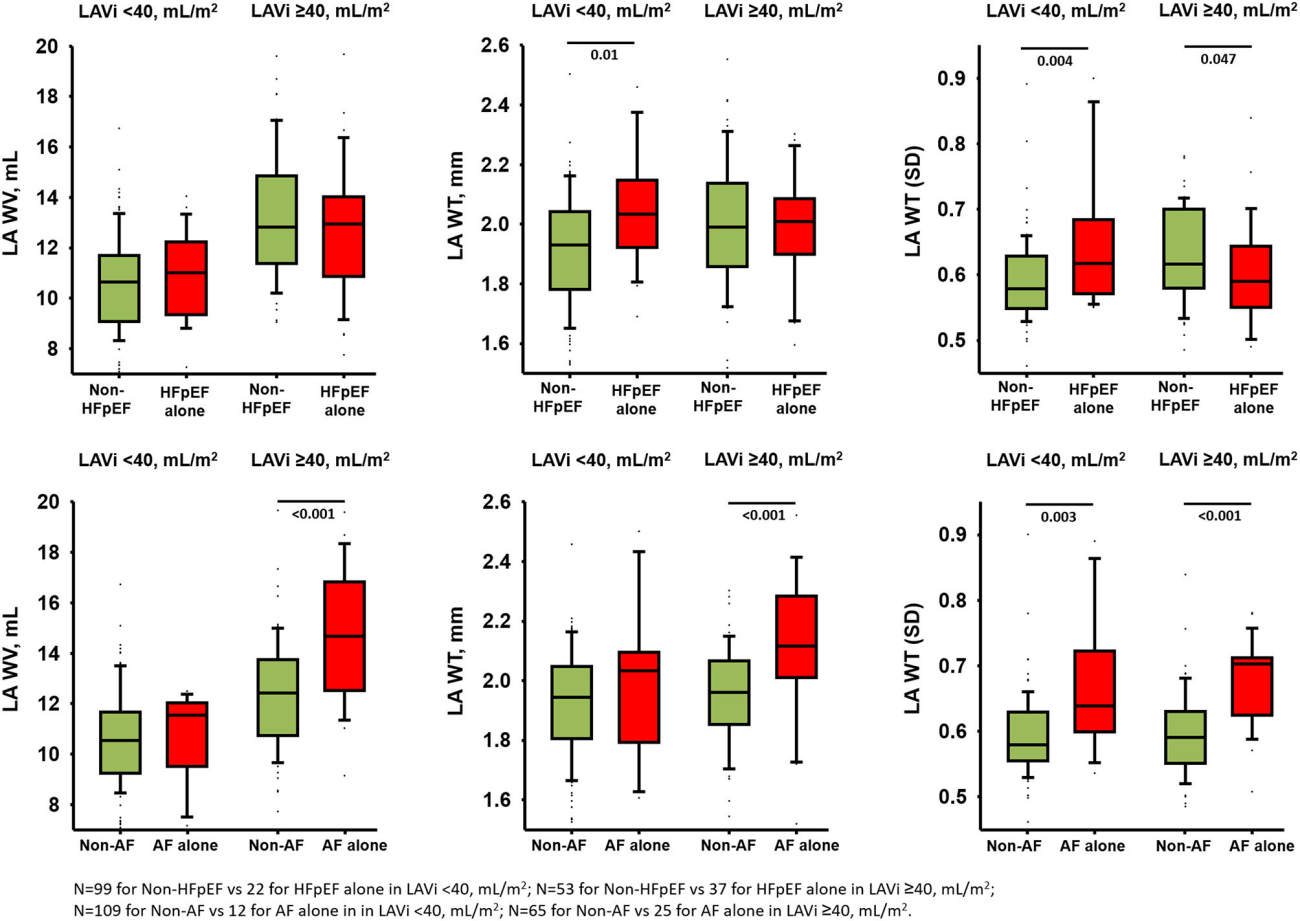
Comparisons of LA wall indices in isolated HFpEF and AF in smaller and larger indexed LA volume after excluding patients with both HFpEF and AF (total *n* = 211).

All LA wall indices were negatively associated with PALS (Figure 2B, beta-coefficients for LAVi, LAWV, LAWT, and LAWT(SD) were −0.43, −0.40, −0.36, and −0.21, respectively; all *p* ≤ 001), indicating the association between the increase of LA wall indices and worsening global LA function (Figure 2B). All correlations were non-linear, as indicated by MIC-r^2^ > 0.1 (Figure 3, Supplementary Figure 1; details in Supplementary Materials). Variable clustering and similarity assessment (Figure 3) showed that LAWV had significant proximity with LAVi; whereas LAWT(SD) tightly coupled with LAWT, did not show significant proximity with any echocardiographic parameters in the dendrogram (Supplementary Figure 2), suggesting the potential value of these measures as novel LA metrics depicting LA remodeling.

### Incremental Value of LA Wall Indices by MDCT in Discrimination of AF vs. HFpEF Alone

After excluding patients with HFpEF+AF (final *n* = 211), the LAWT(SD) demonstrated the highest discriminatory ability for distinguishing isolated AF from isolated HFpEF [optimal cutoff: 0.60, C-statistic: 0.78 (95% CI: 0.70–0.86); sensitivity: 93.8%, specificity: 68.2%] among all LA wall indices (Figure 2A). Besides, isolated AF [adjusted Coef: 0.08 (95% CI: 0.05–0.11), *p* <0.001] rather than isolated HFpEF was independently associated with larger LAWT (SD) after correcting baseline co-variates and E/e'. Remarkably, the trend of differences in LAWT (SD) comparing HFpEF vs. non-HFpEF was flipped after stratified by LAVi; whereas such trend was consistent when comparing AF vs. non-AF after stratified by LAVi, suggesting “diffuse” vs. “patchy” LA wall thickening in HFpEF vs. AF during LA enlargement (Figure 3). Finally, LAWV and LAWT (SD) significantly re-classify isolated AF from control and isolated HFpEF when added to LAVi [continuous net reclassification improvement (NRI): 56.9% (*p* = 0.002) and 71.2% (*p* <0.001)] and PALS [NRI: 42.2% (*p* = 0.02] and 72.7% (*p* <0.001), respectively] (Supplementary Table 3).

## Discussion

To the best of our knowledge, this is the first study to compare LA wall characteristics and function using MDCT method in addition to echocardiography among controls, patients with HFpEF alone, AF alone, and both HFpEF and AF. Overall, increase of each LA wall index was related to worse LA function assessed by strain, with each LA wall index showing distinctive associations with corresponding clinical risk factors. Despite similar LAVi, LAWT, and PALS, patients with AF alone had significantly larger LAWV and LAWT (SD) as compared to the patients with HFpEF alone, even after LA afterload (E/e') taken into account. Furthermore, LAWT (SD) showed the largest C-statistic value in discriminating isolated AF from isolated HFpEF patients and control. Besides, LAWT (SD) did not show significant proximity to any echocardiographic parameter assessing LA, LV function by cluster analysis, suggesting that LAWT (SD) is likely to reflect a novel dimension of LA remodeling.

Although LA remodeling has shown to be a hallmark feature in both HFpEF and AF, no study has explored the distinct LA remodeling patterns in comparison of HFpEF with AF particularly in terms of the LA wall characteristics. Particularly, no studies described LA wall characteristics in patients with HFpEF, while a few studies reported LA wall characteristics in patients with AF with discrepant findings. Nakamura et al. found that the LA wall was thicker in patients with AF than in controls, which was in line with our findings (9). In the same study, patients with paroxysmal AF showed thicker LA wall than those with persistent AF (9). Conversely, Imada et al. found no differences in the LA wall thickness between patients with paroxysmal and those with persistent AF (9, 10). Although the durations and different types AF (i.e., paroxysmal vs. persistent) among the different cohorts might explain the discrepancy in LA wall thickening vs. thinning in patients with AF, but, it is often impossible to pinpoint the exact date of onset of AF in individual patient. On the other hand, these findings indicate that LA wall thickening is a dynamical process with AF progression. We postulated that distinct changes of LA wall may occur with a transition from either AF alone or HFpEF alone to concomitant HFpEF and AF in addition to changes in LA enlargement and dysfunction, which require further validation in future studies.

Similar to patients with AF alone, patients with HFpEF alone also presented with a larger LA wall volume, thicker LA wall, and worse LA global function than the controls. Importantly, despite a similar extent of LA enlargement/dysfunction and a lower LA afterload (i.e., lower E/e', lower BNP), patients with AF alone still manifested significantly larger LAWV and LAWT (SD) than patients with HFpEF alone after multiple adjustments (Table 2), indicating LA afterload might not be the single predominant pathological determinant driving greater wall thickness heterogeneity in isolated AF. These findings support the concept of distinct LA remodeling exist in HFpEF vs. AF. Patients with HFpEF are likely more characterized by “diffuse” LA wall thickening secondary to chronic LA hypertension resulting in increased LA stiffness. Whereas, patients with AF are more characterized by “patchy” LA wall thickening with greater LAWT (SD) irrespective of extent of LA enlargement. These “patchy” LA wall thickening consisting of continuous fibrotic insulations of myo-bundles may further exaggerate LA wall heterogeneity itself and mechanistically contribute to microanatomic re-entry substrates whereby harboring or maintaining AF and set up a vicious cycle of “AF begets AF” (11–13). Nakatani et al. found that LA wall thickness heterogeneity did not differ among patients with paroxysmal and persistent AF, suggesting that “patchy” LA wall thickening as a consistent LA remodeling pattern of AF irrespective of the AF type (14). Notably, these findings are novel since no overlap was found between parameter of LAWT(SD) in current study and other LA indices using comprehensive echocardiography measures. Taken collectively, these unique yet different features of LA wall remodeling support the concept of “diffuse” vs. “patchy” pattern of LA wall thickening between HFpEF vs. AF alone. Besides, when patients with AF are prone to develop concomitant HFpEF, the LA wall will inevitably ensue “diffused” pattern superimposed “patchy” wall thickening during LA size expansion.

**Figure 4:**
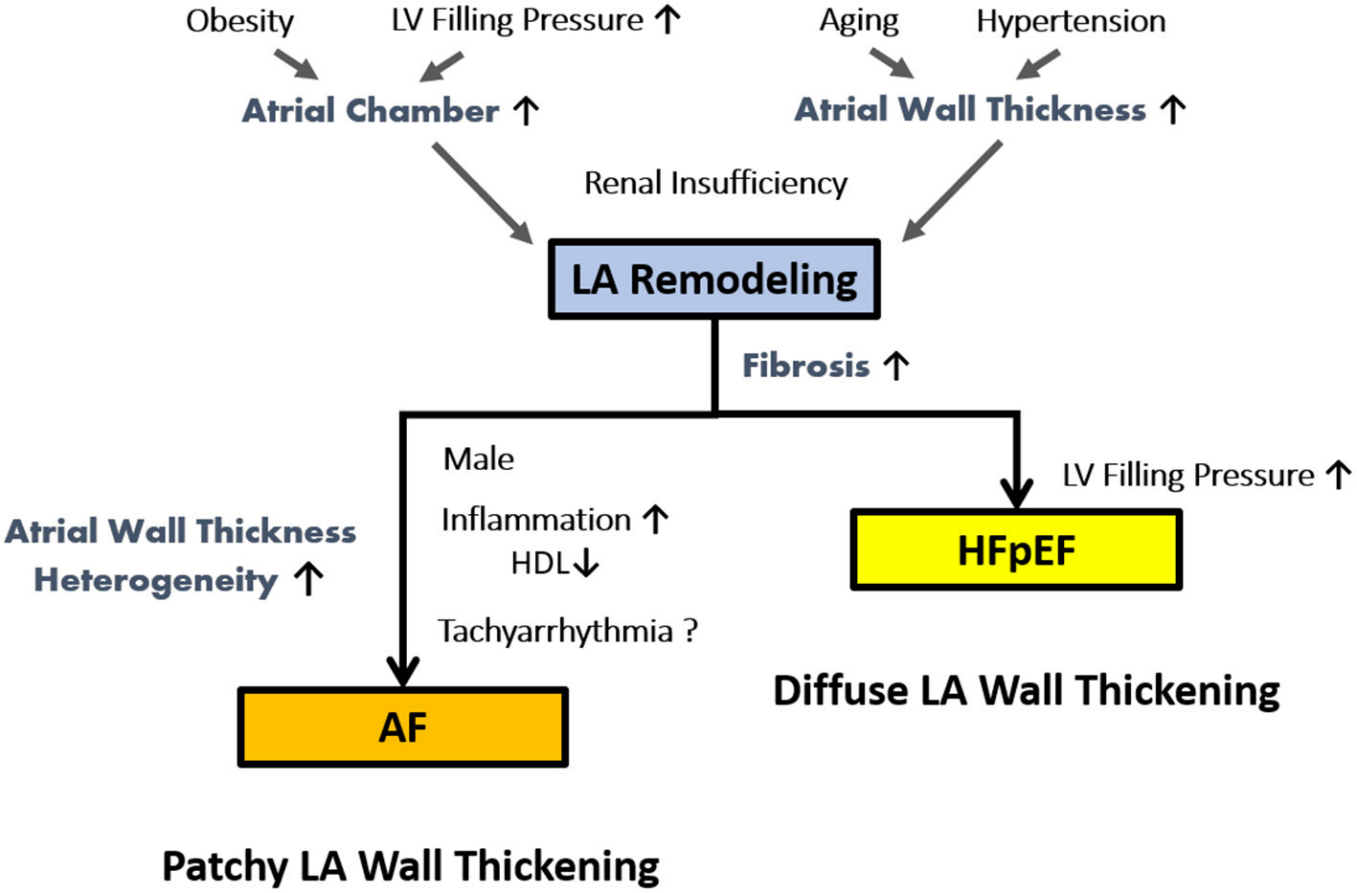
Hypothetical distinctive pathological mechanisms of LA remodeling in AF and HFpEF.

We found that each LA wall index was distinctively associated with corresponding clinical covariates such as aging, obesity, metabolic or renal dysfunction, and LA afterload (E/e'), which heterogeneously promote and amplify specific distinguishable LA wall remodeling patterns (15, 16). We hypothesized that LA wall thickening accompanied by atrial volume expansion, atrial myocyte hypertrophy and interstitial fibrosis is a heterogeneous process resulted from aging, obesity, fluid retention and LA afterload, and further contribute to LA afterload increase itself. During these processes, a distinct LA remodeling pattern with differential LA wall characteristics may occur in parallel with predisposition to HFpEF vs. AF driven by certain gender (male vs. female) or metabolic (such as HDL level) effects together with overlapping risk factors (Figure 4) (11, 13–17). Patients with both AF and HFpEF may present with the worst LA function from both pathological LA remodeling features as compared to the other groups (12), with LA wall changes comprising both diffuse and patchy thickening.

## Limitations

This was a non-randomized, single-center retrospective study with a relatively small number of patients in the HFpEF+AF group as compared to the rest groups. We acknowledged the potential technical limitations of MDCT in discriminating specific, regional anatomical landmarks (for example, the crista terminalis or cava) with averaged LAWT or LAWT (SD) which may indicate a substrate in the pathogenesis of atrial myopathy in AF. Finally, the current study could not establish the definitive mechanism of LA remodeling and the pathological causal relationship between LA wall characteristics and the clinical risk factors in HFpEF and AF. Nonetheless, this is the very first study using MDCT and echocardiography to provide novel insights into LA remodeling in both HFpEF and AF. Finally, as we sought to identify progressive and potentially distinctive LA remodeling patterns in patients manifesting HFpEF or AF when compared to those without (controls), rigorous matching for baseline characters among controls and HFpEF/AF patients were not performed. Our findings warrant further study in other larger prospective cohorts.

## Conclusions

Despite a similar extent of LA enlargement and dysfunction in AF and HFpEF, a larger LAWV and greater LAWT (SD) distinguish AF from HFpEF, suggesting differential mechanisms underlying driving distinct LA remodeling patterns in AF vs. HFpEF.

## Data Availability Statement

The datasets presented in this article are not readily available because of regulations from local Institutional Review Board. Requests to access the datasets should be directed to the corresponding author.

## Ethics Statement

The study protocol was approved by the Institutional Review Board of MacKay Memorial Hospital (19MMHIS213e) as a retrospective research design, written informed consent from patients/participants was waived in this study.

## Author Contributions

J-YK: collected data, analyzed data, and wrote the paper. C-LH and T-HW: conceived, designed the study, and edited the paper. XJ and J-YS: performed the statistical analysis. C-HY and F-PC: acquisition of data, analysis, and interpretation of data. S-HC, P-CC, K-TS, GM, S-CC, and C-HY: conceptual frame work. S-HC, P-CC, K-TS, GM, S-CC, CL, and C-HY: reviewed paper. H-IY: have given final approval of the version to be published. All authors have read and approved the final manuscript.

## Funding

This research was supported by the Ministry of Science and Technology (Taiwan) (grants NSC-101-2314-B-195-020, NSC103-2314-B-010-005-MY3, 103-2314-B-195-001-MY3, 101-2314-B-195-020 –MY1, MOST 103-2314-B-195-006-MY3, and NSC102-2314-B-002-046-MY3, 106-2314-B-195-008 -MY2, 108-2314-B-195-018-MY2, MOST 108-2314-B-195-018-MY2, MOST 109-2314-B-715-008, and MOST 110-2314-B-715-009-MY1), MacKay Memorial Hospital (10271, 10248, 10220, 10253, 10375, 10358, and E-102003), and the Taiwan Foundation for Geriatric Emergency and Critical Care.

## Conflict of Interest

The authors declare that the research was conducted in the absence of any commercial or financial relationships that could be construed as a potential conflict of interest.

## Publisher's Note

All claims expressed in this article are solely those of the authors and do not necessarily represent those of their affiliated organizations, or those of the publisher, the editors and the reviewers. Any product that may be evaluated in this article, or claim that may be made by its manufacturer, is not guaranteed or endorsed by the publisher.
