# Supplementary material for: Insights on Distinct Left Atrial Remodeling Between Atrial Fibrillation and Heart Failure With Preserved Ejection Fraction
Source: Front Cardiovasc Med. 2022 Apr 26;9:857360. doi: 10.3389/fcvm.2022.857360 (PMC9086706; doi:10.3389/fcvm.2022.857360)
Supplement: Supplementary file 1 [file Data_Sheet_1.DOC]

**(Supplemental Materials)**

**Insights on Distinct Left Atrial Remodeling between Atrial Fibrillation and Preserved Ejection Fraction Heart Failure**

**Short Title: LA Wall Characterization in AF versus HFpEF**

**Authors**

Jen-Yuan Kuo, MD,^a,b,c^ Xuanyi Jin, MD,^l^ Jing-Yi Sun, PhD,^d^ Sheng-Hsiung Chang, MD,^a,c^ Po-Ching Chi, MD,^e^ Kuo-Tzu Sung, MD,^a,b,c^ Greta S.P. Mok, PhD,^f^ Chun-Ho Yun, MD, PhD,^b,c,g^ Shun-Chuan Chang, PhD,^h^ Fa-Po Chung, MD, PhD,^i,j^ Ching-Hsiang Yu^b^, Tung-Hsin Wu, PhD,^d,*^ Chung-Lieh Hung, MD, PhD,^a,b,k,*^ Hung-I Yeh, MD, PhD,^a,b,c^ Carolyn S.P. Lam, MD, MBBS, PhD^l^

**Affiliations**

^a^Division of Cardiology, Department of Internal Medicine, MacKay Memorial Hospital, Taipei, Taiwan

^b^Department of Medicine, Mackay Medical College, New Taipei City, Taiwan

^c^Mackay Medicine, Nursing, and Management College, and Taipei, Taiwan

**^d^Department of Biomedical Imaging and Radiological Sciences, National Yang Ming University, Taipei, Taiwan**

^e^Division of Cardiology, Department of Internal Medicine, Taoyuan General Hospital, Taoyuan, Taiwan

^f^Biomedical Imaging Laboratory, Department of Electrical and Computer Engineering, Faculty of Science and Technology, University of Macau, Macau SAR, China

^g^Department of Radiology, **Mackay Memorial Hospital, Taipei, Taiwan**

^h^**Holistic Education Center, Mackay Medical College, New Taipei City, Taiwan**

**^i^**Institute of Clinical Medicine and Cardiovascular Research Center, National Yang-Ming University**, Taipei, Taiwan**

^j^Heart Rhythm Center and Division of Cardiology, Department of Medicine, Taipei Veterans General Hospital, **Taipei, Taiwan**

^k^Institute of Biomedical Sciences, Mackay Medical College, Taipei, Taiwan;

^l^National Heart Centre Singapore, and Duke-National University of Singapore

*Address for Correspondence

Tung-Hsin Wu, PhD., Department of Biomedical Imaging and Radiological Sciences, National Yang Ming University, Taipei, Taiwan. Address: 155 Li-Nong St., Sec. 2, Taipei 112, Taiwan.

Tel: 886-2-28267061; E-mail: tung@ym.edu.tw

Chung-Lieh Hung, MD., MSc., PhD., Division of Cardiology, MacKay Memorial Hospital, Taipei, Taiwan. Address: 92, Sec 2, Chung Shan North Road, Taipei 10449, Taiwan.

Tel: 886-2-2543-3535 ext. 2456 Fax: 886-2-2543-3535 ext. 2459; E-mail: jotaro3791@gmail.com

**Methods**

**Cardiac Multi-detector Computed Tomography (MDCT) Image Acquisition Protocol**

The CT scanner used in the study was a dual-source, high resolution CT system (Siemens Medical Systems, Forchheim, Germany). The scan protocol included a retrospective electrocardiography-gating, 120-kVp tube voltage, 320 reference mA with an automatic exposure control, and pitch of 0.28. The raw data was reconstructed by a slice thickness of 0.75 mm, construction increment of 0.4 mm, and common cardiac kernel (B26f) associated with a field-of-view of 160 mm, 512 × 512 matrix, and resolution of 0.31 mm per pixel. The scan parameters for clinical patients were automatically adjusted with a pitch from 0.28 to 0.38, according to the heart beats of each patient. The range of the z-axis for each scan was from a level of 1 cm below the carina to the dome of the diaphragm. A pre-scan test bolus study was performed using an injection of 10 mL of nonionic iodinated contrast medium (Iopamiro, Bracco Industria Chimicas p.a., Milano, Italy) followed by 20 mL of normal saline delivered by a power injector at a rate of 5 mL/sec to determine the peak enhancement in the ascending aorta. Second, the injection of the remaining 50 mL of nonionic iodinated contrast medium was begun and delivered by a power injector at a rate of 5 mL/sec, and the scans were then commenced with a delay equal to the time to the peak enhancement plus 8 seconds. The subsequent LA wall analysis was performed using reconstructed CT images obtained during the LA diastolic phase (i.e., ventricular systole), i.e., between 30% and 40% of the R-R interval phases.

**Program for MDCT-based LA Wall-Mapping and LA wall characteristics in Current Study**

The previously-described method of the LA wall-mapping program has been tested and its accuracy compared to a phantom and further validated in a porcine model (1). In brief, the LA wall-mapping program included a set of semi-automatic procedures that could process and analyze the global LA wall thickness and visualize the map in a coplanar plane. It also allowed for a simultaneous localization with cardiac CT images and consisted of five procedures. The first procedure, termed segmentation and LA delineation, was the only one needing manual annotation. Its purpose was to separate the pulmonary vein (PV) and automatically set the isocenter of the LA chamber, based on previously published work, with truncations of adequate PVs performed by an adequate clipping point 10 mm from the ostium (2).

In procedure 2, the LA chamber containing contrast medium was automatically delineated using the region growing method through dilation from the isocenter. In combination with the Sobel method, the inner boundary of the LA could be delineated automatically. By integrating the total number of voxels contained within the inner surfaces, the LA chamber volume (LAV) could be semi-automatically assessed and quantified. After a dilation algorithm was performed on the inner boundary, the Otsu threshold method was used to find the Hounsfield unit threshold between the LA wall and surrounding soft tissue, to delineate the outer boundary (procedure 3), with the entire LA surface area segmented as in the previous method (3). The algorithm then converged to the boundary between the LA wall and surrounding soft tissue to define the boundary as the outer boundary. Procedure 4 was the wall thickness calculation. The shortest distance between the outer boundaries and inner surfaces was calculated using the Euclidean distance method, with representative LA wall thickness (LA WT) defined as averaged distance within whole LA region. LA wall volume (LA WV) was derived by integrating the total number of voxels of LA tissue which rendered total LA volume contained within the outer boundaries minus the chamber volume contained by the inner surfaces. Procedure 5 was the image-space transformation. The purpose was to expand the wall thickness data obtained from procedure 4 from the CT images into a three-dimensional (3D) LA shell view and to visualize the data as a projected map.
 The expanded image space was called the two-dimensional (2D) mapping space. The boundary of the left atrial appendage (LAA) and delineation of the mitral plane was identified as in the previous literature and was not included in the LA wall metrics assessments (4). Finally, the index of the LA wall thickness heterogeneity (LA WT[SD]) was calculated as a measure of the degree of LA WT dispersion (expressed as standard deviation [SD]) within whole LA region generated from LA wall-mapping program. Measurements of the LA wall volume, wall thickness, and wall thickness heterogeneity were obtained in all study subjects. The schematic workflow of our imaging processing, transformation, and LA wall mapping is further shown in Figure 2. Among a total of 249 (42 AF, 207 non-AF) datasets initially eligible to enter into the current study, finally 229 had a sufficient image quality to render the final LA morphological indices analyzable (96% successful rate).

**Similarity Assessment and Variable Clustering of the MDCT-based LA wall characteristics and the use of Correlation Explorations**

A hierarchical clustering analysis organizes closely-related objects or variables into a dendrogram whose branches are the desired clusters (Figure 4A), with the approximation estimate of the unbiased probability (AU) value or p-value reported. A clustering model identifies the similarity metrics of possible coupled, closest objects or parameters, and further segregates them into groups by the average linkage, forming a dissimilarity matrix as a heat map. The aim was to re-cluster the relevant parameters at different levels. The greater the p-value, the greater the probability that the grouping condition was true. The dissimilarity matrix was then visualized as a phenomap of the Pearson’s correlation (green-black-red). Each row represented an individual patient and each column corresponded to a single clinical variable of the key clinical variables including all Echo-based parameters, MDCT-based LA wall metrics, and the baseline age, gender, body mass index (BMI), lipid profiles, estimated glomerular filtration rate (eGFR), levels of inflammatory biomarkers such as the C-reactive protein (CRP), and critical clinical medical information. The independent column on the right side is shown as the diagnosis of the patient, including Control group (in yellow), HFpEF (in green), AF (in blue), and AF + HFpEF (in red). The AU probability (red numbers) and bootstrap probability (BP, green numbers) of the column dendrogram as measurements of the certainty for clusters are calculated and displayed. The AU values >95%, framed in blue (95-99%) and orange (100%), were considered statistically significant.

Linear and non-linear correlations between the MDCT-based LA wall metrics and baseline characters or various diastolic parameters were tested using Pearson’s correlation coefficient (r) or the maximal information coefficient (MIC) method. MIC is a new method for detecting non-linear correlations. By exploring the difference between the absolute values of the MIC and squared Pearson correlation coefficient (r^2^) (MIC – r^2^), a marker of the non-linear correlation can be created. MIC – r^2^ denotes a non-linear pattern and then can be subsequently confirmed by visual inspection of the correlation plots between two variables.

**Results**

**Findings from the heat maps between the MDCT-based LA wall characteristics and echocardiography based key LA/LV Indices**

All correlations were of non-linear nature, as indicated by MIC-r^2^ > 0.1.

Supplemental Figure 1 illustrates the heat maps of the linear Pearson’s correlations overlapped with a dot scatter plot matrix for the first step involving the visual inspection of the significant associations among the LA wall characteristics, baseline covariates, and diastolic parameters in the uni-variate models (A). Direct correlations are demonstrated in shades of blue (the darker the color, the more it approves a correlation coefficient of 0.5), and inverse correlations are demonstrated in shades of red (the darker the color, the more it approves a correlation coefficient of -0.5). Dot plots are in gray to show the distributional relationships between the variables (A). A squared correlation coefficient (r^2^) (B), MIC (C), and the difference between the MIC and r^2^ (MIC – r^2^, the correlation would be nonlinear when the difference was bigger than 0.1) (D) were demonstrated for the MDCT-based LA wall metrics that most optimally represented the correlates with conventional echocardiography-derived parameters (such as the LVMi, E/A ratio, TDI-e’, TDI-s’, LV filling E/e’, and TR velocity [TRV]) and key deformational measurements (such as the GLS, global LA strain [PALS], and the strain rates [reservoir, conduit, and booster pump phases]) as non-linear patterns.

A larger LAVi and LA WV had a better linear correlation with a higher TRV (Pearson r: 0.49, 0.43, MIC=0.23, 16; both p<0.001), lower TDI-e’ (Pearson r: -0.20, -0.15, MIC=0.29, 0.39, p<0.001 and 0.016), higher E/e’ (Pearson r: 0.38, 0.31, MIC=0.26, 0.23, both p<0.001), lower LAEF (Pearson r: -0.39, -0.33, MIC=0.21, 0.27, both p<0.001), worsened GLS (Pearson r: 0.33, 0.31, MIC=0.24, 0.25, both p<0.001), more impaired PALS (both Pearson r: -0.40, MIC=0.24, 0.21, both p<0.001), lower LA reservoir (Pearson r: -0.40, -0.42, MIC=0.22, 0.16, both p<0.001), and conduit (Pearson r: 0.34, 0.37, MIC=0.32, 0.23, both p<0.001), as well as an impaired LA booster pump function (Pearson r: 0.39, 0.38, MIC=0.26, 0.23, both p<0.001).

Likewise, a greater LA WV had a mild-to-modest linear correlation with a higher TRV (Pearson r: 0.31, MIC=0.23, p<0.001), lower TDI-e’ (Pearson r: -0.11, MIC=0.27, p=0.08), higher E/e’ (Pearson r: 0.20, MIC=0.30, p=0.002), lower LAEF (Pearson r: -0.16, MIC=0.37, both p=0.012), worsened GLS (Pearson r: 0.27, MIC=0.25, p<0.001), impaired PALS (Pearson r: -0.35, MIC=0.25, p<0.001), lower LA reservoir (Pearson r: -0.39, MIC=0.25, p<0.001), and conduit (Pearson r: 0.34, MIC=0.21, p<0.001), as well as an LA booster pump functional decline (Pearson r: 0.36, MIC=0.24, p<0.001). Instead, a larger LA WT(SD) had a mild linear correlation with a higher TRV (Pearson r: 0.22, MIC=0.28, p<0.001), non-significant linear relationship with the TDI-e’ (Pearson r: -0.07, MIC=0.31, p=0.30), higher E/e’ (Pearson r: 0.16, MIC=0.24, p=0.013), lower LAEF (Pearson r: -0.18, MIC=0.31, both p=0.005), worsened GLS (Pearson r: 0.16, MIC=0.27, p=0.014), impaired PALS (Pearson r: -0.19, MIC=0.33, p=0.002), lower LA reservoir (Pearson r: -0.18, MIC=0.24, p=0.005), and conduit (Pearson r: 0.11, MIC=0.31, p=0.08), as well as an LA booster pump functional decline (Pearson r: 0.21, MIC=0.31, p=0.001). Overall, the LA WT and LA WT(SD) showed that the majority of the differences between the MIC and r^2^ were more than 0.1 and near 0.3 (MIC- r^2^ >0.1) but presenting with lower Pearson’s linear correlations, indicating intermediate non-linear correlations among the MDCT-based LA WT/WT(SD) and echocardiography-determined ventricular and atrial functional parameters, which were underestimated using the conventional methods such as Pearson’s correlation coefficient.

***Variable clustering and similarity assessment***

Our clustering analysis showed that LA WV had significant proximity with LAVi within the clustering dendrograms (approximately unbiased [AU] probability>95%, suggesting a high statistical overlap) (Figure 4B). Instead, LA WT(SD) coupled with LA WT without significant proximity with other echocardiography parameters within the clustering dendrograms (all AU probability <95%), suggesting that while LA WV indicated LA size in general, LA WT(SD) provided information beyond traditional LA indices by echocardiography.

**Supplemental Table 1. Variability and reproducibility of MDCT-based LA wall characteristics (n=30)**

| **LA Indices** | **Intra-observer** | | **Inter-observer** | |
| --- | --- | --- | --- | --- |
| **Metrics for Reproducibility** | **ICC** | **COV** | **ICC** | **COV** |
| LAV, mL * | 0.97 | 7.4% | 0.97 | 8.2% |
| LA WV, mL | 0.98 | 3.7% | 0.96 | 5.1% |
| LA WT, mm | 0.97 | 2.8% | 0.96 | 3.2% |
| LA WT(SD) | 0.96 | 3.4% | 0.95 | 4.5% |

* variability information for maximal LA volume measures based on MDCT method.

LAV=LA volume (maximal value) by MDCT, LA WV=LA wall volume, LA WT= LA wall thickness, LA WT(SD)=LA wall thickness heterogeneity.

**Supplemental Table 2. Comparisons of baseline variables after propensity matching from isolated HFpEF and control groups vs. isolated AF group (1:1 matching as n=37 vs. n=37)**

| **Metabolic Score Categories** | **AF Alone (n=37)** | **Control + HFpEF Alone (n=37)** | **P Value** |
| --- | --- | --- | --- |
| **Baseline Demographics** |  |  |  |
| Age, years | 59.70 (10.33) | 63.97 (10.97) | 0.089 |
| Female sex, (%) | 15 (40.5%) | 15 (40.5%) | 1 |
| BMI, kg/m^2^ | 25.83 (4.24) | 25.94 (3.21) | 0.898 |
| Hypertension, (%) | 30 (81,1) | 27 (73,0) | 0.407 |
| Diabetes, (%) | 17 (45.9) | 13 (35.1) | 0.344 |
| Hyperlipidemia, (%) | 16 (43.2) | 15 (40.5) | 0.814 |
| Coronary artery disease, (%) | 6 (16.2) | 5 (13.5) | 0.744 |
| eGFR, mL/min/1.73m^2^ | 79.04 (25.40) | 77.24 (24.12) | 0.755 |
| E/e’ | 9.75 (3.78) | 11.36 (5.29) | 0.139 |

**Supplemental Table 3. Continuous net reclassification improvement [NRI] for MDCT-based LA wall characteristics in discriminating isolated AF when added to LAVi and PALS (total n=211).**

| **Improvement of MDCT-based LA wall characteristics vs LAVi** | | |
| --- | --- | --- |
| **NRI table** | Improvement in | Improvement in |
|  | LAVi | LA WV |
| **Non-AF** | 63.60% | 36.40% |
| **AF (Isolated)** | 35.10% | 64.90% |
| Category-free NRI: 56.9%, p=0.002 | | |
| **NRI table** | Improvement in | Improvement in |
|  | LAVi | LA WT |
| **Non-AF** | 52.60% | 47.40% |
| **AF (Isolated)** | 45.90% | 54.10% |
| Category-free NRI: 13.3%, p=0.48 | | |
| **NRI table** | Improvement in | Improvement in |
|  | LAVi | LA WT(SD) |
| **Non-AF** | 65.30% | 34.70% |
| **AF (Isolated)** | 29.70% | 70.30% |
| Category-free NRI: 71.2%, p<0.001 | | |
|  | | |
| **Improvement of MDCT-based LA wall characteristics vs PALS** | | |
| **NRI table** | Improvement in | Improvement in |
|  | PALS | LA WV |
| **Non-AF** | 87.20% | 74.00% |
| **AF (Isolated)** | 12.80% | 26.00% |
| Category-free NRI: 42.2%, p=0.02 | | |
| **NRI table** | Improvement in | Improvement in |
|  | PALS | LA WT |
| **Non-AF** | 86.60% | 76.90% |
| **AF (Isolated)** | 13.40% | 23.10% |
| Category-free NRI: 32.6%, p=0.10 | | |
| **NRI table** | Improvement in | Improvement in |
|  | PALS | LA WT(SD) |
| **Non-AF** | 90.80% | 68.40% |
| **AF (Isolated)** | 9.20% | 31.60% |
| Category-free NRI: 72.7%, p<0.001 | | |

**Supplemental Figure 1. Heat maps showing the correlations among MDCT-based LA wall metrics and echocardiography-based key LA/LV indices.**

**
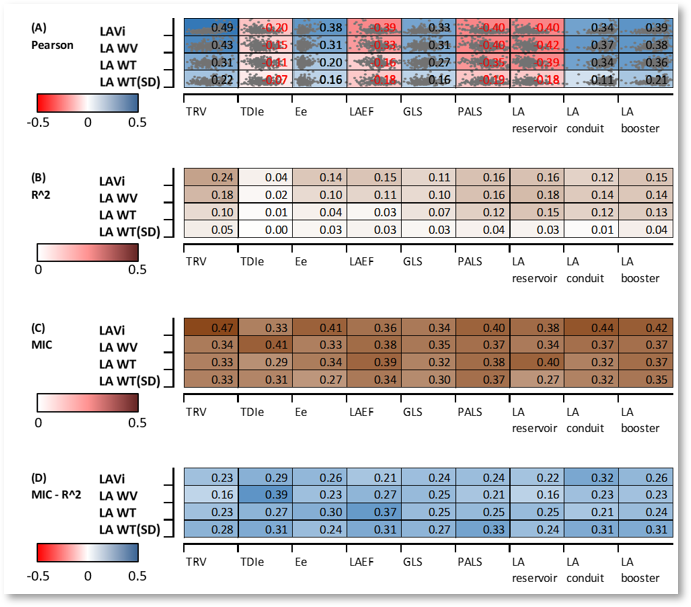
**

Heat maps of the correlation illustrations between the MDCT and echocardiography-based structural/functional variables were displayed regarding the following: (A) Heat maps overlapping with the correlations using correlation dot plots, B: Squared correlation coefficient (r^2^), (C) Maximal Information Coefficient (MIC), and (D) the difference between the MIC and r^2^, the higher these differences (e.g. > 0.1), the greater the possibility of non-linear associations.

GLS= global longitudinal strain, LA=left atrial, LAEF= left atrial ejection fraction, LAVi= left atrium volume index, LV= left ventricular, LVEF= left ventricular ejection fraction, MDCT= multi-detector computed tomography, PALS= global longitudinal LA strain, TDI-e’= mitral annular early relaxation velocity by tissue Doppler imaging, E/e’= ratio of the early transmitral hemodynamic Doppler E velocity divided by TDI-e’ (average), TR-v= TR velocity

**Supplemental Figure 2. The workflow of the correlations and dissimilarity matrix visualization as a precision phenomapping of the MDCT-based LA wall indices and echocardiography-based parameters**

**
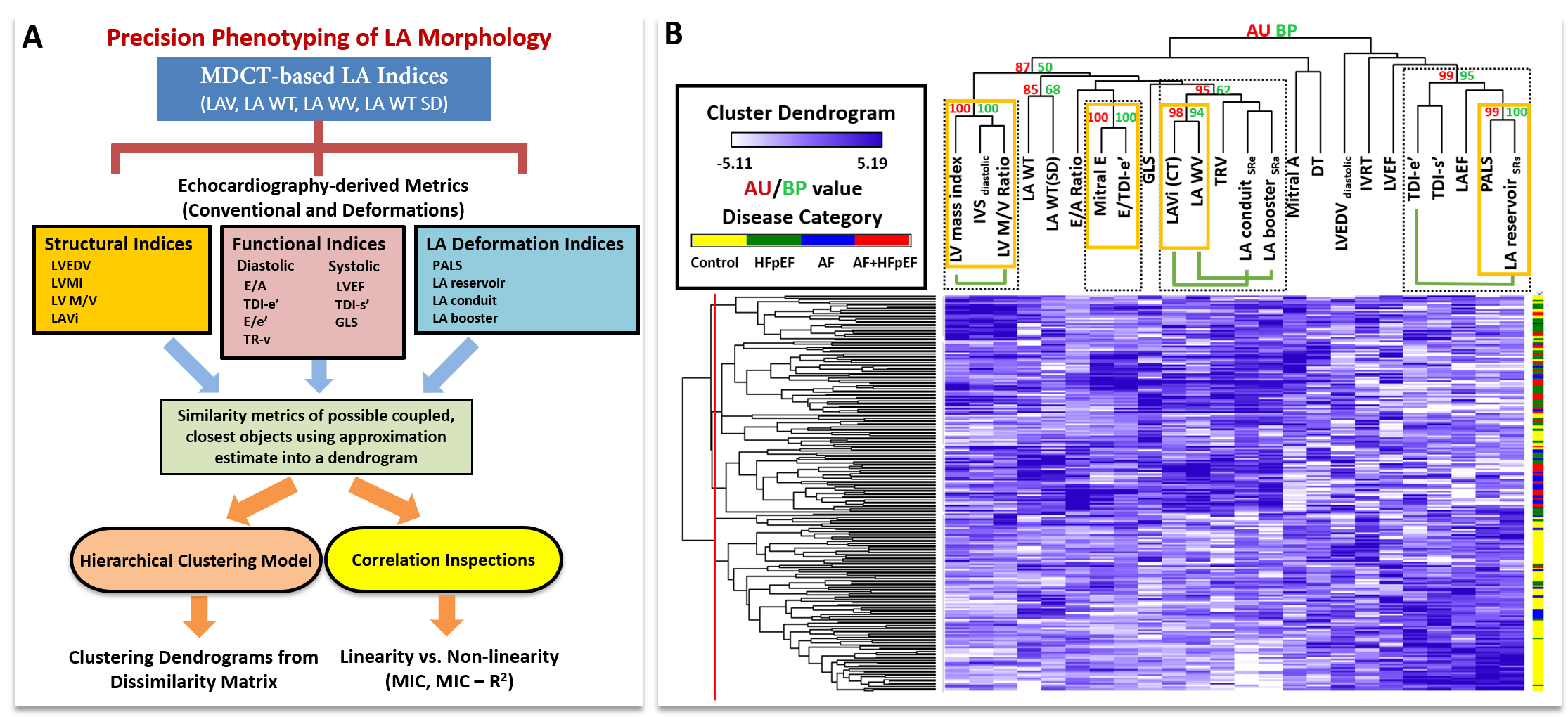
**

The workflow of the correlations and similarity explorations with variable clustering of the MDCT-based LA wall indices constructed by using a hierarchical clustering model (**A**). Extracted MDCT-derived LA wall were assessed for the proximity to echocardiographic parameters evaluating LV and LA structural and functional parameters forming a dissimilarity matrix as a heat map (**B**). For figure B, the approximately unbiased probability (AU, red numbers) and bootstrap probability (BP, green numbers) of the column dendrogram were calculated and demonstrated. The AU values >95% were framed in red and considered statistically significant. AU= approximately unbiased, BP= bootstrap probability.

All abbreviations as Table 1, 2 and Figure 1**.**

**References**

1. Sun JY, Yun CH, Mok GSP, et al. Left atrium wall-mapping application for wall thickness visualization. Sci Rep 2018;8:4169.
2. Tobon-Gomez C, Geers A, et al. Benchmark for algorithms segmenting the left atrium from 3D CT and MRI datasets. IEEE Trans Med Imaging 2015;34:1460–73.
3. Koppert MMJ, Rongen PMJ, Prokop M, ter Haar Romeny BM, van Assen HC. Cardiac left atrium CT image segmentation for ablation guidance. In: Biomedical Imaging: From Nano to Macro, 2010 IEEE International Symposium on 2010 Apr 14 (pp. 480-483). IEEE.
4. Schmidt B, Ernst S, Ouyang F, et al. External and endoluminal analysis of left atrial anatomy and the pulmonary veins in three-dimensional reconstructions of magnetic resonance angiography: the full insight from inside. J Cardiovasc Electrophysiol 2006;17:957–64.
